# Induced pluripotent stem cells from a transgenic minipig model of Huntington's disease reveal early metabolic changes

**DOI:** 10.1242/dmm.052585

**Published:** 2026-05-11

**Authors:** Irena Rysankova, David Sekac, Hana Hansikova, Katerina Vodickova Kepkova, Petr Vodicka, Michaela Vaskovicova, Marie Altmanova, Stefan Juhas, Jana Juhasova, Eliska Taborska, Jiri Klempir, Jan Motlik, Jiri Klima, Lars Eide, Zdenka Ellederova

**Affiliations:** ^1^ Department of Neurology and Centre of Clinical Neuroscience, First Faculty of Medicine, Charles University in Prague and General University Hospital in Prague, 120 00 Prague, Czech Republic; ^2^ Laboratory of Cell Regeneration and Plasticity, Institute of Animal Physiology and Genetics of the Czech Academy of Science, 277 21 Libechov, Czech Republic; ^3^ Department of Cell Biology, Faculty of Science, Charles University in Prague, 128 00 Prague, Czech Republic; ^4^ Laboratory for Study of Mitochondrial Disorders, Department of Paediatrics and Adolescent Medicine, First Faculty of Medicine, Charles University and General University Hospital in Prague, 12108 Prague 2, Czech Republic; ^5^ Laboratory of Applied Proteome Analyses, Institute of Animal Physiology and Genetic of the Czech Academy of Sciences, 277 21 Libechov, Czech Republic; ^6^ Laboratory of DNA integrity, Institute of Animal Physiology and Genetics of the Czech Academy of Science, 277 21 Libechov, Czech Republic; ^7^ Laboratory of Fish Genetics, Institute of Animal Physiology and Genetics of the Czech Academy of Science, 277 21 Libechov, Czech Republic; ^8^ Laboratory of Biotransformation, Institute of Microbiology of the Czech Academy of Sciences, 142 00 Prague, Czech Republic; ^9^ Department of Medical Biochemistry, Institute of Clinical Medicine, University of Oslo and Oslo University Hospital, 0372 Oslo, Norway

**Keywords:** Huntington's disease, Transgenic minipig model, Induced pluripotent stem cells, Mitochondria, Gene expression, DNA damage

## Abstract

Huntington's disease (HD) is a neurodegenerative autosomal dominant hereditary disease caused by a CAG triplet repeat expansion mutation in the gene encoding the huntingtin (HTT) protein. The main feature of HD is the loss of striatal neurons, accompanied by metabolic and transcriptional alterations in both neural and peripheral tissues.

Induced pluripotent stem cells (iPSCs) derived from a transgenic HD (TgHD) minipig model expressing a mutant HTT construct were generated to investigate early metabolic, antioxidant and DNA integrity changes associated with HD development. Gene expression analysis showed increased expression of vascular endothelial growth factor (*VEGF*), pyruvate dehydrogenase kinase 1 (*PDK1*) and glutamine-oxaloacetic transaminase 1 (*GOT1*), implying early metabolic alteration in TgHD iPSCs. Moreover, upregulated FANCD2/FANCI-associated nuclease 1 (*FAN1*) expression indicated genotoxic stress linked to early HD development. These findings suggest metabolic shifts and putative genotoxic events in the pluripotent stem cell state of the TgHD model and point to early effect of the HD mutation. The model may be suitable for evaluating potential cell therapy and *in vitro* differentiation of iPSCs to neurons and other cells affected in HD.

## Teaser

**Summary:** Induced pluripotent stem cells from transgenic Huntington's disease minipigs display disrupted pyruvate metabolism, accompanied by elevated *VEGF* and transglutaminase levels, and emphasise the link between *FAN1* and Huntington's disease pathology.

## INTRODUCTION

Huntington's disease (HD) is a fatal, autosomal dominant neurodegenerative disorder with no known cure. It affects approximately 3 individuals per 100,000 globally, although prevalence varies significantly by region – ranging from as low as 0.40 per 100,000 in Asian populations to as high as 5.7 per 100,000 in Europe, North America and Australia. HD is caused by pathological expansion of a CAG trinucleotide repeat in exon 1 of the huntingtin (*HTT*) gene on chromosome 4p16.3. This mutation leads to the production of mutant HTT (mHTT) protein with an extended polyglutamine tract. The number of CAG triplets is thought to be one of several factors influencing age at onset and progression of the disease (Andresen et al., 2007). The expression of mHTT within the central nervous system leads to a progressive degeneration of GABAergic medium spiny neurons in the striatum, as well as neuronal loss across multiple subcortical and cortical regions, contributing to widespread neuroanatomical and functional deficits (Rosas et al., 2008). In addition to its central nervous system manifestations, a number of primarily metabolic changes in peripheral tissues are also described. The expanded CAG tract can fold into a hairpin structure, which drives somatic expansion with age. It has been proposed that this is an important trigger of the onset of disease (Swami et al., 2009).

HD is clinically characterised by a combination of motor, psychiatric and cognitive symptoms that progressively worsen over time and significantly impact daily functioning and quality of life (Jensen et al., 1998; Johnson et al., 2007; Marshall et al., 2007; Ross et al., 2014; Verny et al., 2007). Weight loss during normocaloric or even hypercaloric intake is also a well-documented phenomenon in HD, yet its underlying pathophysiological mechanisms remain unclear. Compared to unaffected controls, patients with HD exhibit elevated energy expenditure, which increases with disease duration but does not correlate with the severity of motor or functional impairment (Aziz et al., 2010). Although increased energy expenditure due to involuntary motor activity may contribute, growing evidence points to a broader dysregulation of energy homeostasis at both cellular and endocrine levels. In the early stages of HD, decreased levels of branched-chain amino acids and insulin-like growth factor 1 (IGF-1) have been reported, suggesting a systemic metabolic defect (Mochel et al., 2007). Additional findings include reduced cerebral glucose metabolism in the striatum (Powers et al., 2007), decreased ATP levels in HD fibroblasts, impaired mitochondrial activity and elevated production of mitochondrial reactive oxygen species (Jędrak et al., 2018), causing oxidative stress and DNA damage. Other mitochondrial abnormalities observed in HD include disrupted mitochondrial trafficking (Li et al., 2010), calcium homeostasis dysfunction (Lim et al., 2008), and altered mitochondrial metabolism and changes in mitochondria morphology that are present in symptomatic patients and also in pre-manifest HD gene carriers (Lopes et al., 2022).

In 2006, Yamanaka and Takahashi published a paper that marked a huge technological breakthrough in stem cell research (Takahashi and Yamanaka, 2006). They reprogrammed mouse somatic cells into cell lines similar to embryonic stem cells by using four defined transcriptional factors: octamer-binding transcription factor 4 (OCT4), SRY (sex determining region Y)-box 2 (SOX2), Krüppel-like factor 4 (KLF4) and cellular myelocytomatosis oncogene (c-MYC; also known as MYC) (Takahashi and Yamanaka, 2006). A year later, human somatic cells were reprogrammed with the same result (Takahashi et al., 2007; Yu et al., 2007).

This advancement opened new possibilities for modelling human diseases *in vitro*. Induced pluripotent stem cells (iPSCs) derived from patients or disease models retain the genetic background of the donor. They can be differentiated into various cell types, making them powerful tools for studying disease mechanisms and phenotypes. In this context, the iPSCs from patients with HD have been used to uncover disease-specific impairments (HD iPSC Consortium, 2012; Świtońska et al., 2019).

iPSCs from patients with HD exhibit naturally reduced mitochondrial respiration and lower mitochondrial membrane potential (Folmes et al., 2011; Panopoulos et al., 2012; Varum et al., 2011; Zhang et al., 2012; Zhu et al., 2010). This reduction is reflected in increased glycolysis, whereby the metabolites are involved in pluripotency maintenance by histone acetylation or upregulation of the pentose phosphate pathway. These processes ensure nucleotide synthesis and a reduced NADPH pool necessary for antioxidant defence (Folmes et al., 2011; Moussaieff et al., 2015; Varum et al., 2011). Subsequent studies have revealed the importance of hypoxia-inducible factor 1α (HIF1A) as a modulator of metabolic shift by upregulation of pyruvate dehydrogenase kinase 1-3 (PDK1-3) and the M2 isoform of pyruvate kinase (Prigione et al., 2014).

The expression of mHTT causes lower ATP levels in iPSCs and iPSC-derived neurons from patients with HD than in controls, together with decreased mitochondrial respiratory capacity (Kedaigle et al., 2021; Lopes et al., 2020). As a compensatory response, basal glycolysis and glycolytic capacity are higher in iPSCs from patients with HD than in controls. However, in iPSC-derived neurons, there is evidence of lower glycolytic capacity with lowered glycolytic enzyme expression (Kedaigle et al., 2021; Lopes et al., 2020).

Apart from disturbed metabolism, oxidative stress-related proteins, together with higher levels of hydrogen peroxide, are present in iPSCs from patients with HD (Chae et al., 2012; Lopes et al., 2020). A broader overview of phenotypic features of iPSCs from patients with HD and neuronal progenitor cells is summarised in Kaye et al. (2022). These studies are indicative of early metabolic alteration in HD.

In agreement with these data, an iPSC model derived from a large animal would be relevant and highly additive to a human model and could assess metabolic changes and other phenotypic features caused by mHTT. This would be a preliminary step to preclinical testing before studies *in vivo*, biomarker discovery or development of new therapies for HD.

Here, we generated iPSC lines from embryonic fibroblasts of a transgenic HD (TgHD) minipig model expressing the N-terminal fragment of human HTT (N548), with 124 glutamine repeats encoded by mixed CAA–CAG sequences (Baxa et al., 2013). The TgHD minipig was originally established by lentiviral-mediated expression of human mHTT under the control of the human HTT promoter and exhibits a slowly progressive, age-dependent HD-like phenotype, characterised by functional locomotor decline, mitochondrial DNA (mtDNA) damage, altered mtDNA copy number, metabolic dysregulation and progressive neuropathology (Askeland et al., 2018).

By deriving TgHD iPSCs, this study aims to establish a cellular platform that can be directly integrated with the corresponding *in vivo* model and used to investigate metabolic, antioxidant and DNA integrity status in order to identify early mHTT-driven cellular abnormalities occurring in the absence of somatic CAG expansion, thereby facilitating mechanistic links between cellular and animal phenotypes in HD.

## RESULTS AND DISCUSSION

### Generation of porcine iPSCs from wild-type and TgHD embryonal fibroblasts

**Fig. 1.**
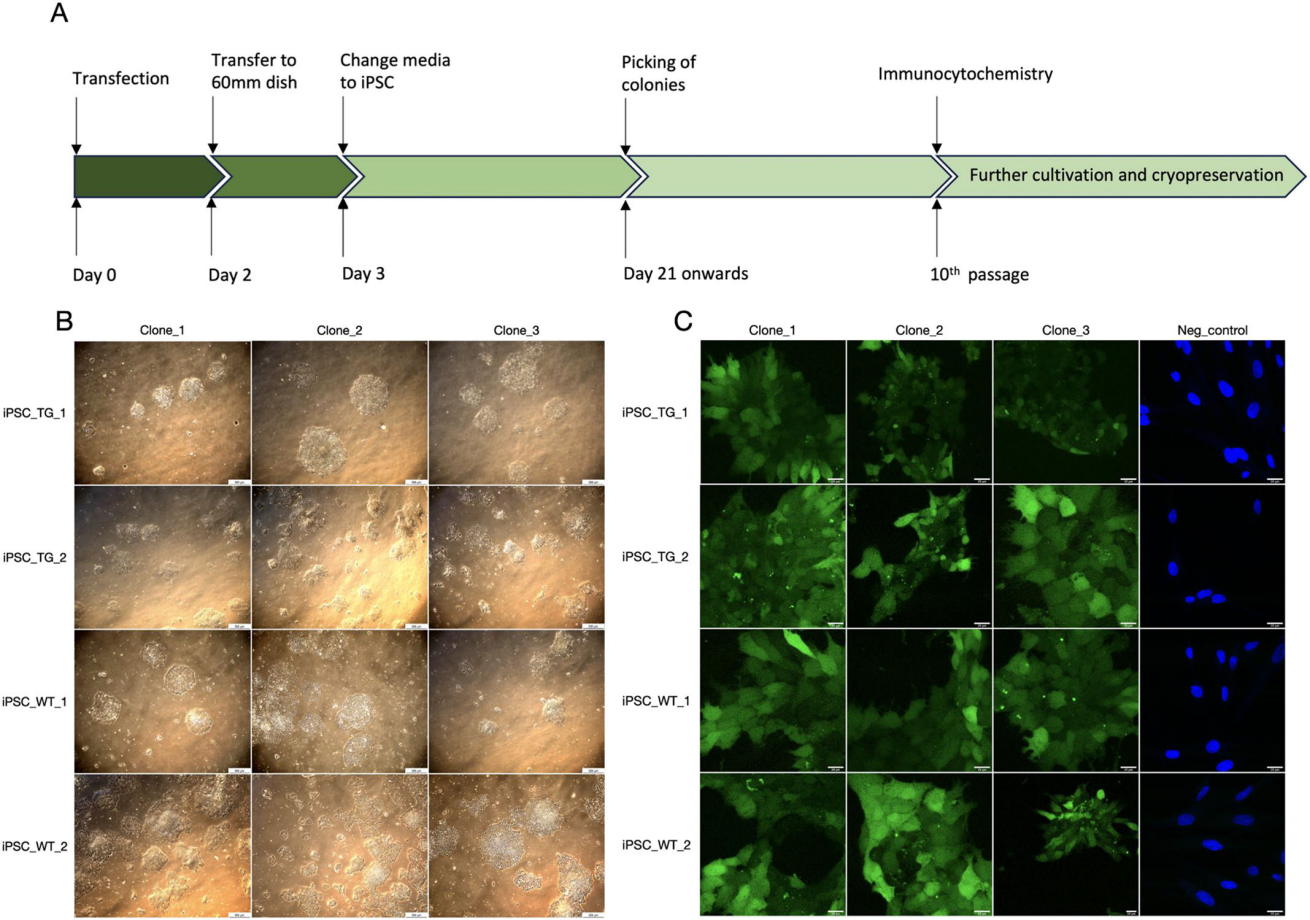
**Reprogramming of primary embryonal fibroblasts to induced pluripotent stem cells (iPSCs).** (A) Timeline of reprogramming. Primary embryonal fibroblasts (PEFs) were transfected with piggyBac plasmid containing reprogramming factors OCT4, SOX2, KLF4, c-MYC and LIN28 together with a transposase. Transfected cells were transferred to a 60 mm Petri dishes after 48 h. The next day, the cultivation medium was replaced with iPSC medium supplemented with 10 ng/ml bFGF, 10 ng/ml LIF, 10 μM RepSox, 10 μM CHIR99021 and 1 μM PD0325901. The medium was changed daily, and transfected cells remained undisturbed until iPSC colonies formed. These colonies were then manually picked and expanded. *n*=2 WT PEFs, *n*=2 TgHD PEFs. The experiment was repeated twice. Representative staining of the TgHD iPSC line is shown. *n*=6, TgHD iPSCs. (B) Brightfield images of iPSC colonies. Representative brightfield images of iPSC colonies at passage 13. *n*=6 WT iPSCs, *n*=6 TgHD iPSCs. The experiment was repeated twice. Scale bars: 500 µm. (C) Alkaline phosphatase staining in iPSC colonies. Alkaline phosphatase staining was performed at passage 13 and was positive in both transgenic Huntington's disease (TgHD) and wild-type (WT) iPSCs. Primary fibroblasts derived from the same donor animals were used as a negative control. The nuclei were stained with Hoechst 33342. *n*=6 WT iPSCs, *n*=6 TgHD iPSCs. The experiment was repeated twice. Scale bars: 20 µm.

Fibroblasts isolated from wild-type (WT) minipigs and TgHD minipigs, which express the N-terminal part of human mHTT in all cells, were used to generate iPSCs and further characterised (Figs S1-S3, Table S5). First colonies with typical embryonic stem cell-like morphology started to appear 3-6 weeks after transfection of reprogramming factors (Fig. 1). These compact colonies displayed well-defined sharp edges and contained round cells with large nuclei and reduced cytoplasm, characteristics typical of pluripotent cells.

**Fig. 2.**
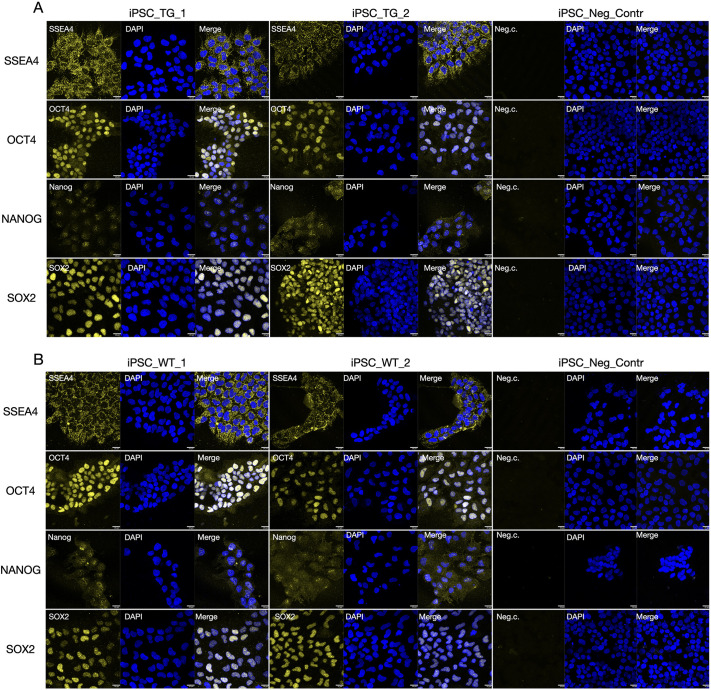
**Immunocytochemical characterization of pluripotency marker expression in TgHD and WT iPSCs.** (A) Positive expression of the pluripotency markers SSEA4, OCT4, NANOG and SOX2 in TgHD iPSCs. The TgHD iPSC line 1, without primary antibody incubation and followed by a fluorophore-conjugated secondary antibody, was used as a negative control. Representative staining of the TgHD iPSC line is shown. *n*=6, TgHD iPSCs. (B) Positive expression of the pluripotency markers SSEA4, OCT4, NANOG and SOX2 in WT iPSCs. The WT iPSC line 1, without primary antibody incubation, followed by a fluorophore-conjugated secondary antibody, was used as a negative control. Representative staining of the WT iPSC line is shown. *n*=6, TgHD iPSCs. For both panels, the iPSCs were seeded on 1% Geltrex-coated LabTek chamber slides at passage 13. After reaching 70-80% confluency, the iPSCs were fixed and stained using primary antibodies against the indicated markers, followed by fluorophore-conjugated secondary antibody. Nuclei were counterstained with DAPI. The staining was performed in duplicates for each iPSC line. The same exposure setup was used for each marker across all samples. Scale bars: 20 µm. See Materials and Methods for detailed antibody information and imaging conditions.

**Fig. 3.**
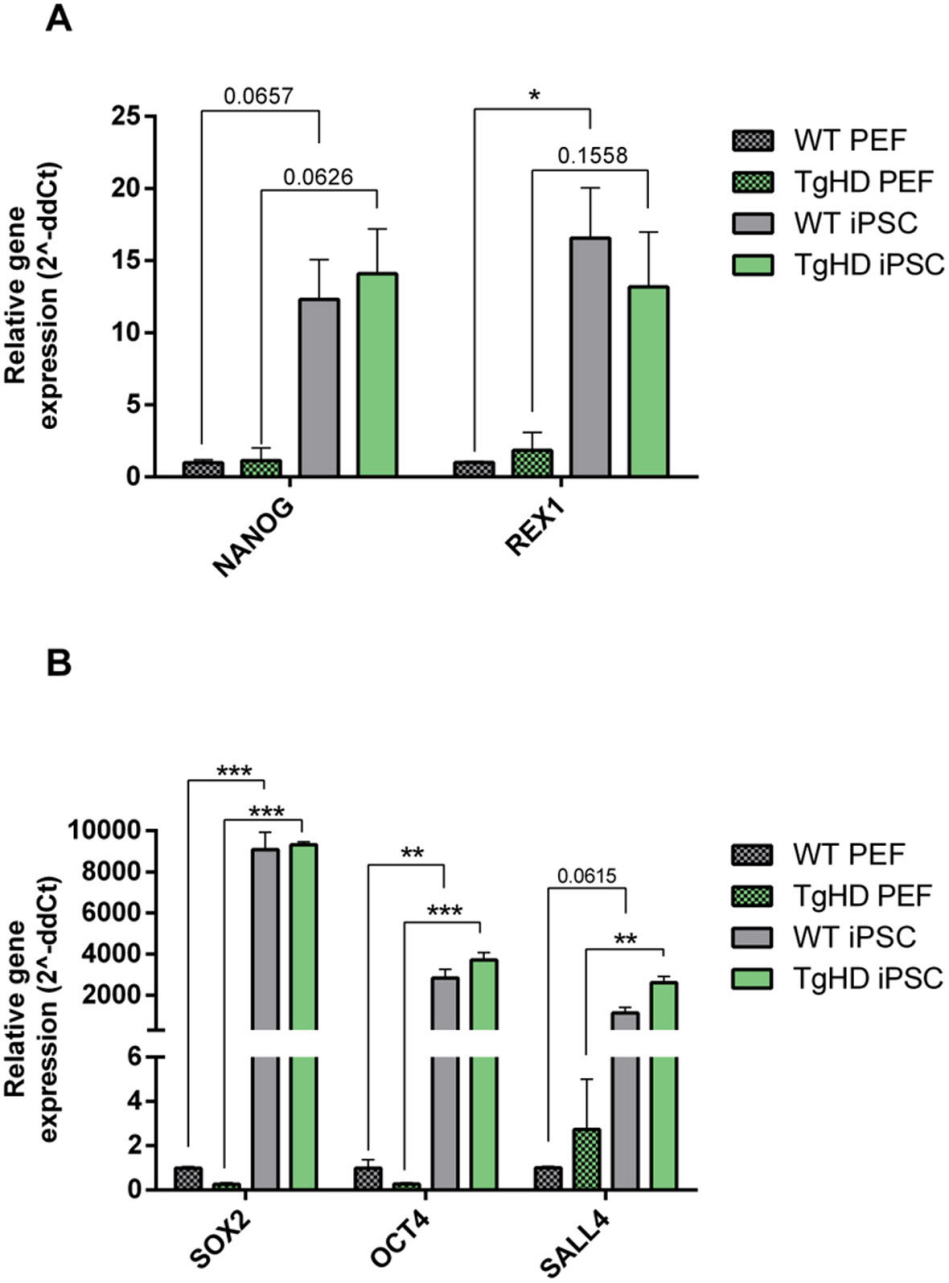
**Markers of pluripotency.** (A) Comparison of endogenous *NANOG* and *REX1* expression between WT iPSCs and TgHD iPSCs with that in WT PEFs and TgHD PEFs, normalised to the average of WT PEFs. *P*-values are as follows: *NANOG* (*P*=0.0657, WT PEFs versus WT iPSCs; *P*=0.0626, TgHD PEFs versus TgHD iPSCs), *REX1* (*P=0.0494, WT PEFs versus WT iPSCs; *P*=0.1558, TgHD PEFs versus TgHD iPSCs). (B) Comparison of endogenous *SOX2*, *OCT4* and *SALL4* expression between iPSCs and PEFs. *P*-values are as follows: *SOX2* (***P=0.0004, WT PEFs versus WT iPSCs; ***P<0.0001, TgHD PEFs versus TgHD iPSCs), *OCT4* (**P=0.0026, WT PEFs versus WT iPSCs; ***P=0.0006, TgHD PEFs versus TgHD iPSCs), *SALL4* (*P*=0.0615, WT PEFs versus WT iPSCs; **P=0.0038, TgHD PEFs versus TgHD iPSCs). Measurements were performed in a single technical replicate, with each sample loaded in triplicate. The final results were calculated as the mean value of these three analytical replicates. Student's *t*-test with Welch's correction. iPSCs: *n*=6, WT; *n*=6, TgHD. PEFs: *n*=2, WT; *n*=2, TgHD. Error bars indicate s.e.m.

Immunocytochemistry at passage 13 showed detectable expression of the pluripotency markers OCT4, SOX2, stage-specific embryonic antigen-4 (SSEA4) and Nanog homebox (NANOG) in iPSCs derived from both TgHD and WT fibroblasts (Fig. 2A,B). The exogenous reprogramming factors were not excised by the transposase enzyme; therefore, we subsequently evaluated the expression of endogenous porcine *NANOG*, *OCT4*, zinc finger protein 42 (*REX1*), spalt like transcription factor 4 (*SALL4*) and *SOX2* using real-time quantitative PCR (qPCR) analysis of iPSC lines compared to primary embryonal fibroblasts (Fig. 3). Although *NANOG* expression was statistically higher in iPSCs than in porcine embryonal fibroblasts at mRNA level, NANOG protein level and localisation showed weak signal in the nuclei of WT and TgHD iPSCs, probably caused by low antibody affinity to *Sus scrofa* species (Fig. 3). The differentiation potential of iPSC colonies was then tested by induction in endoderm, mesoderm and ectoderm. The ability of the iPSCs to differentiate into all three germ layers was confirmed (Fig. S4).

### Increased VEGF expression with a subtle change in glycolytic gene expression

To explore early metabolic alterations in TgHD iPSCs, we first analysed the expression of key metabolic regulators, including sirtuins (SIRT1, SIRT2), HIF1A and vascular endothelial growth factor (VEGF). Both sirtuins have been implicated in HD pathogenesis, whereby reduction of SIRT1 contributes to disease progression, while inhibition of SIRT2 leads to neuroprotection (Donmez and Outeiro, 2013). Neuroinflammation and vascular defects with elevated levels of VEGF have also been described in the brain and plasma of patients with HD and R6/2 mice, a model of HD (Chang et al., 2015; Hsiao et al., 2015). VEGF, regulated by HIF1A, plays an essential role in the metabolic shift from oxidative respiration to anaerobic glycolysis (Luo et al., 2011; Pfäfflin et al., 2006) and was, therefore, included in our analysis. We also assessed the expression of the RE1-silencing transcription factor (REST), which is dysregulated in HD and can alter the expression of genes containing neuron-restrictive silencer element (NRSE), for example brain-derived neurotrophic factor (*BDNF*) (Zuccato et al., 2003).

**Fig. 4.**
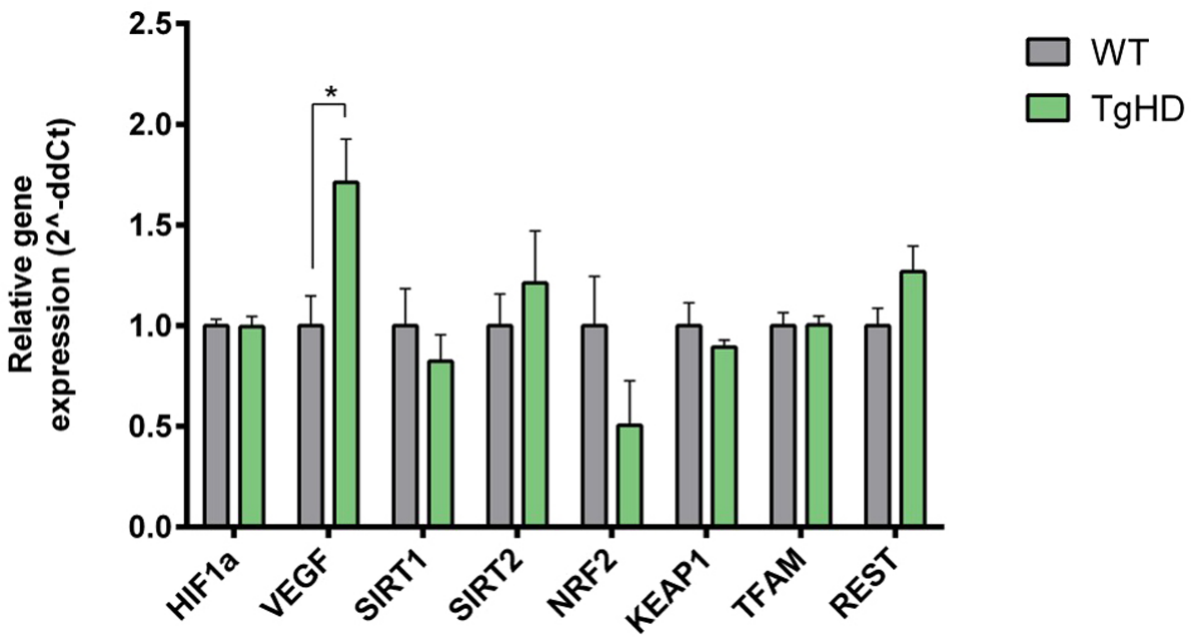
**Expression of regulatory genes.** Relative expression of selected regulatory genes, normalised to the average for WT iPSCs. We observed increased expression of *VEGF* in TgHD iPSCs (*P=0.0323). Measurements were performed in a single technical replicate, with each sample loaded in triplicate. The final results were calculated as the mean value of these three analytical replicates. Student's *t*-test with Welch's correction. *n*=6, WT; *n*=6, TgHD. Error bars indicate s.e.m.

Next, we determined the expression of genes participating in metabolism shift and antioxidative defence: nuclear factor erythroid 2-related factor 2 (*NRF2*), its negative regulator kelch-like ECH-associated protein 1 (*KEAP1*) and transcriptional factor of mitochondria (*TFAM*) (Hawkins et al., 2016; Itoh et al., 1999). Our results showed a significant increase in *VEGF* expression in TgHD iPSCs compared to that in WT iPSCs, with no difference in *HIF1A* expression (Fig. 4). These findings indicate increased anaerobic glycolysis caused by VEGF. Following this, we examined the expression of glycolytic pathway genes (Fig. S5). Although the changes were not statistically significant, TgHD iPSCs showed a trend toward upregulation of hexokinase 2 (*HK2*) (*P*=0.1833) and glyceraldehyde 3-phosphate dehydrogenase (*GAPDH*) (*P*=0.0555), and downregulation of pyruvate kinase (*PKM*) (*P*=0.2180), relative to WT iPSCs, indicating early metabolic dysregulation in TgHD iPSCs.

### Increased expression of pyruvate dehydrogenase kinases and decreased pyruvate dehydrogenase activity in TgHD iPSCs

To follow up on the glycolytic status, the expression levels of regulators of the pyruvate dehydrogenase (PDH) complex, the inactivating pyruvate dehydrogenase kinases (PDK1-4) (Korotchkina and Patel, 2001), as well as the activating pyruvate dehydrogenase phosphatase (PDP1) (Rardin et al., 2009), were determined.

**Fig. 5.**
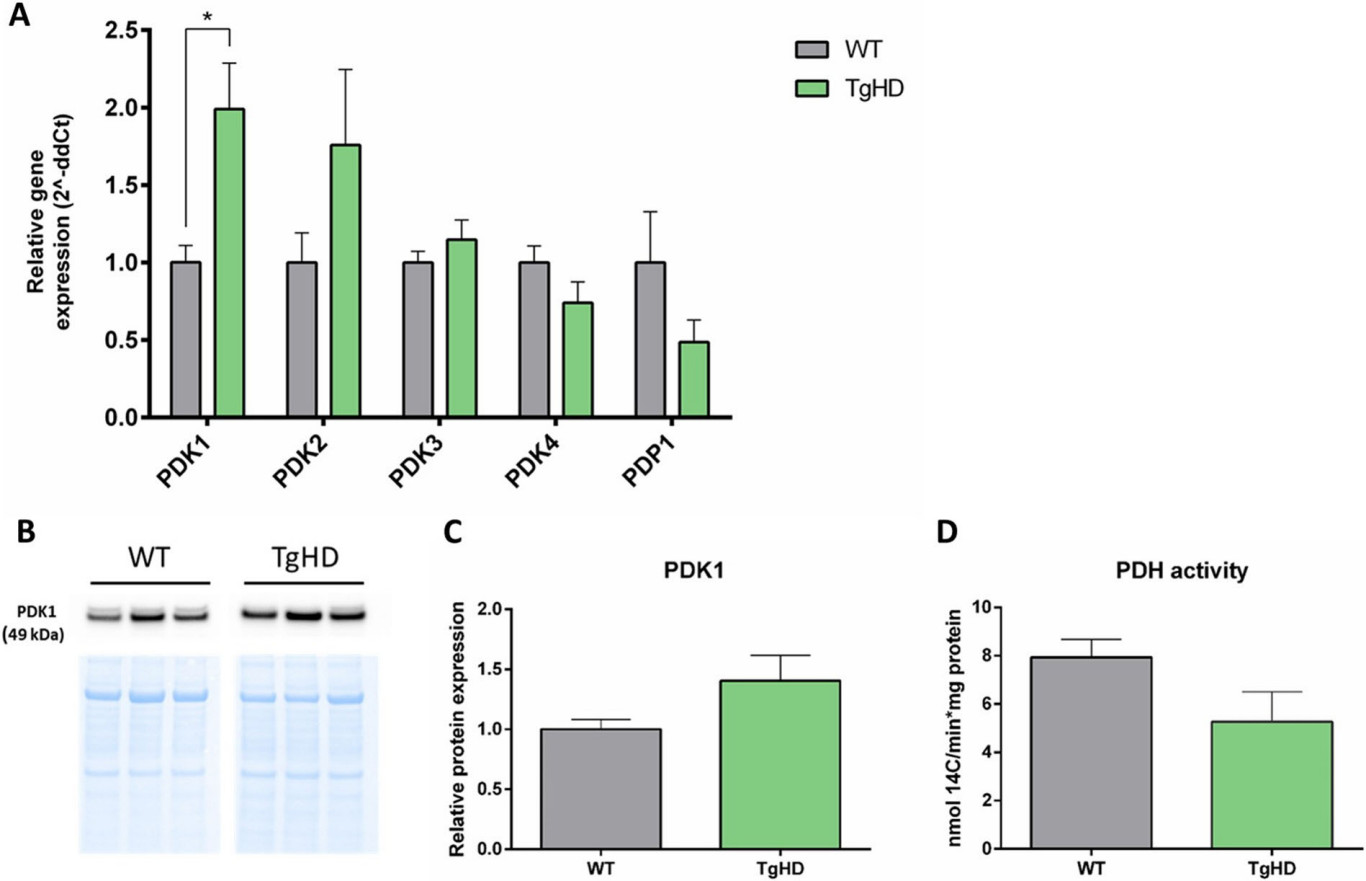
**Expression of *PDK1* and PDH activity.** (A) Elevated expression of PDKs was observed in TgHD iPSCs, normalised to WT iPSCs, which was statistically significant only for *PDK1* (*P=0.02). Measurements were performed in a single technical replicate, with each sample loaded in triplicate. The final results were calculated as the mean value of these three analytical replicates. Student's *t*-test with Welch's correction. *n*=6, WT; *n*=6, TgHD. (B) Representative images of western blot analysis of PDK1 and Ponceau S staining in iPSCs. *n*=3, WT; *n*=3, TgHD. (C) A slight increase in PDK1 protein in TgHD iPSCs relative to that in WT iPSCs was observed (*P*=0.1189). Measurements were performed in a single technical replicate. Student's *t*-test with Welch's correction. *n*=6, WT; *n*=6, TgHD. (D) We also observed a tendency for decreased PDH activity normalised to protein in TgHD iPSCs (*P*=0.0940). Measurements were performed in a single technical replicate, with each sample loaded in triplicate. The final results were calculated as the mean value of these three analytical replicates. Student's *t*-test with Welch's correction. *n*=6, WT; *n*=6, TgHD. Error bars indicate s.e.m.

The results showed a significant increase in *PDK1* expression in TgHD iPSCs, accompanied by a trend toward increased *PDK2* expression and decreased *PDP1* levels (Fig. 5A). Results from western blot analyses displayed similar trends at the protein level (Fig. 5B,C). Both PDK isoforms are expressed in brain structures, muscles and various other tissues (Uhlén et al., 2015). Consequently, these early changes can explain the affected brain and muscle function observed in patients with HD. To validate the cellular effect of these genes, we investigated the activity of PDH. Although not statistically significant, there was a clear trend towards lowering of PDH activity in TgHD iPSCs compared to that in WT iPSCs (Fig. 5D), which is in line with the results obtained from the human iPSC model of HD (Lopes et al., 2020). Lowered PDH activity was also observed in the striatum of postmortem patients with HD (Sorbi et al., 1983).

### Changes in the expression of Krebs cycle-related genes in TgHD iPSCs

**Fig. 6.**
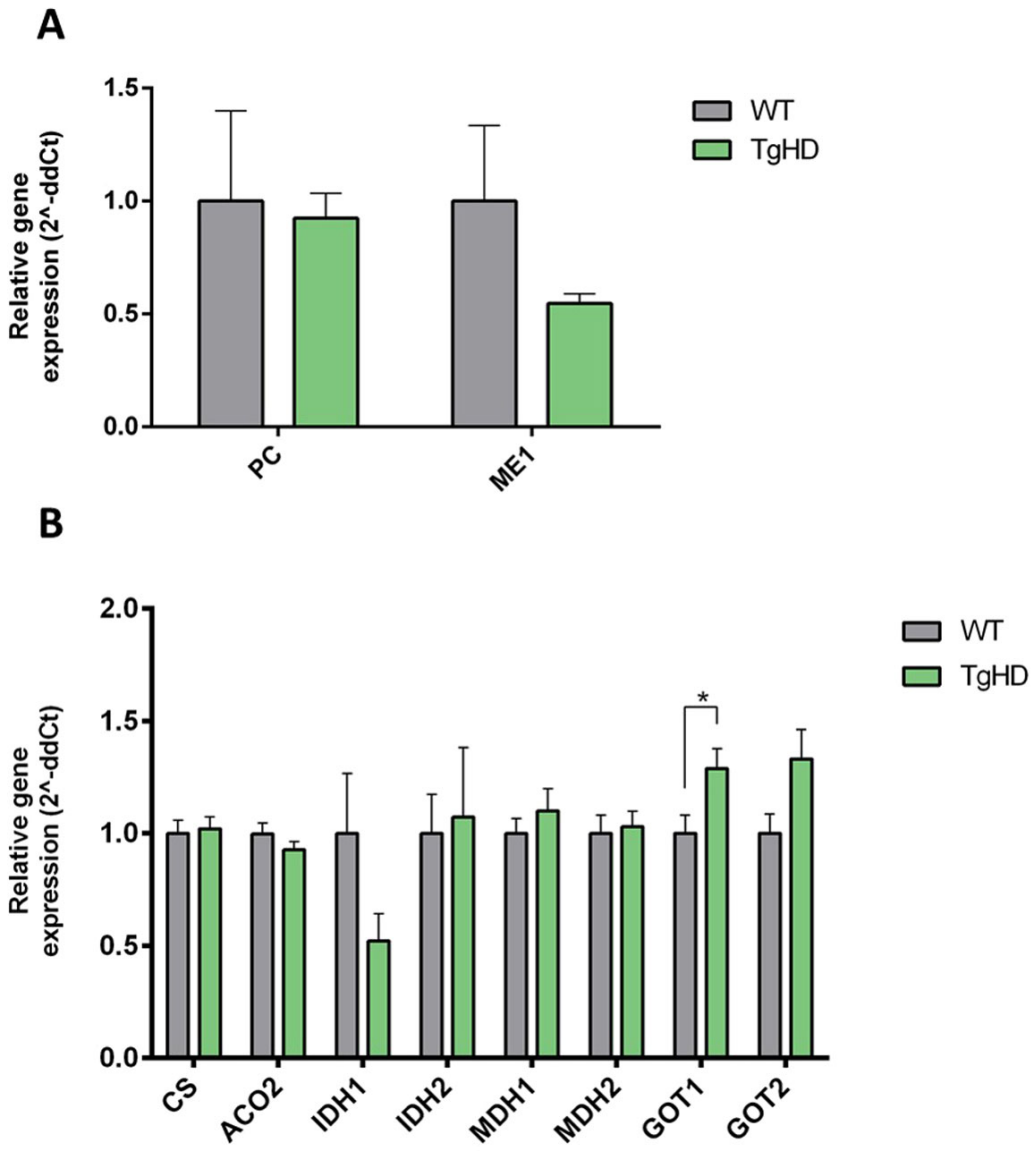
**Expression of *PC*, *ME1* and the expression of Krebs cycle-related genes.** (A) The graph shows the relative mRNA expression of *PC* and a tendency for decreased *ME1* (*P*=0.2047) in TgHD iPSCs, normalised to WT iPSC. (B) Significantly increased expression of *GOT1* (*P=0.0376), together with a non-significant increase in expression of *GOT2* (*P*=0.0730) and a tendency for decreased expression of *IDH1* (*P*=0.1531), was observed in TgHD iPSCs, normalised to WT iPSCs. *n*=6, WT; *n*=6, TgHD. Measurements were performed in a single technical replicate, with each sample loaded in triplicate. The final results were calculated as the mean value of these three analytical replicates. Student's *t*-test with Welch's correction. Error bars indicate s.e.m.

Pyruvate carboxylase (PC) feeds pyruvate into the Krebs cycle via oxaloacetate, while malic enzyme 1 (ME1) generates pyruvate from malate, concurrently reducing NADP^+^ to NADPH (Murai et al., 2017). The tendency for downregulated *ME1* (Fig. 6A) is indicative of reduced capacity to restore pyruvate levels, in line with the above-mentioned results. The lowered *ME1* levels may reflect a limited need for an increased NADPH pool as a co-factor for anabolic reaction or antioxidant enzymes (Del Prado et al., 2024). The same pattern was observed in other NADPH-producing enzymes, such as G6PD (Fig. S3) and *IDH1* (Fig. 6B).

Next, the expression of Krebs-cycle enzymes, citrate synthase (*CS*) and aconitase 2 (*ACO2*), together with other related enzymes, was measured. As shown in Fig. 6B, elevated glutamic-oxaloacetic transaminase 1 and 2 (*GOT1/2*) expression was observed in the TgHD iPSCs compared to WT iPSCs. Both enzymes participate in malate–aspartate shuttle to transfer NADH to mitochondria, on the cytoplasmatic and mitochondrial side, respectively (Peng et al., 2024). These results support increased energetic demand of TgHD iPSCs in a situation in which pyruvate oxidation is reduced.

**Fig. 7.**
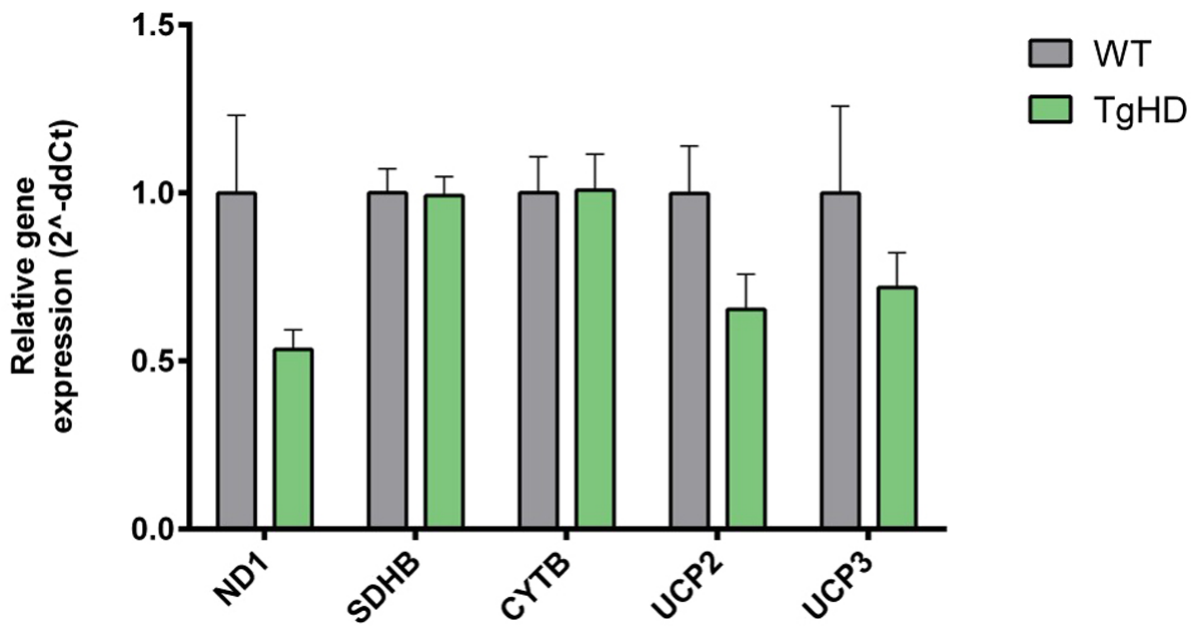
**mRNA expression of mitochondrial complex subunits and uncoupling proteins.** Graph shows lowered expression of mRNA for the complex I subunit (*ND1*) (*P*=0.1015) and unchanged expression of mRNA for complex II subunit (*SDHB*) and complex III subunit (*CYTB*) in TgHD iPSCs normalised to WT iPSCs. The TgHD iPSCs also show decreased *UCP2* (*P*=0.0612) expression in comparison to WT iPSCs (*n*=6, WT; *n*=6, WT). Measurements were performed in a single technical replicate, with each sample loaded in triplicate. The final results were calculated as the mean value of these three analytical replicates. Student's *t*-test with Welch's correction. Error bars indicate s.e.m.

Finally, the mRNA expression of mitochondrial complex subunits [NADH dehydrogenase subunit 1 (*ND1*), succinate dehydrogenase complex iron sulfur subunit B (*SDHB*) and cytochrome B (*CYTB*)], together with uncoupling proteins 2 and 3 (*UCP2*/*3*), was analysed to estimate mitochondrial energetic status. Only a non-significant decrease in complex I subunit *ND1* and *UCP2* in TgHD iPSCs was observed (Fig. 7). The protein levels of ND1 showed a similar tendency to being reduced (*P*=0.1979; Fig. S6). In a human iPSC model of HD, ND1 is elevated but UCP2 is decreased (Lopes et al., 2020), which is consistent with our results. Taken together, these results indicate absence of oxidative stress and reduced mitochondrial respiration without compromised respiratory efficacy in TgHD iPSCs.

### Increased FANCD2/FANCI-associated nuclease 1 expression in TgHD iPSCs without DNA damage

Because PDH activity can respond to metabolic alterations in a DNA repair-dependent manner (Scheffler et al., 2018), we evaluated the expression of DNA repair glycosylases [neil-like DNA glycosylase (NEIL)2, NEIL3, 8-oxoguanine glycosylase (OGG1)] that initiate the repair of modified or oxidised DNA bases. Markers of oxidative stress markers and disrupted antioxidative defence have been observed in brains from patients with HD (Browne et al., 1999; Sorolla et al., 2008). Altered expression of DNA glycosylases has also been described in peripheral blood mononuclear cells of patients with HD (Askeland et al., 2018) and TgHD fibroblasts (Smatlikova et al., 2019).

Additionally, we measured the expression of mitochondrial superoxide dismutase 2 (SOD2), an antioxidant enzyme, along with that of FANCD2/FANCI-associated nuclease 1 (FAN1) and polymerase beta (POLB), both of which are involved in DNA repair (Goold et al., 2019). The importance of FAN1 expression in iPSCs was described in 125Q human iPSCs (Donaldson et al., 2022), fibroblasts from patients with HD and iPSCs-derived astrocytes, in which it has a protective effect against CAG expansion (Cheng et al., 2023 preprint).

**Fig. 8.**
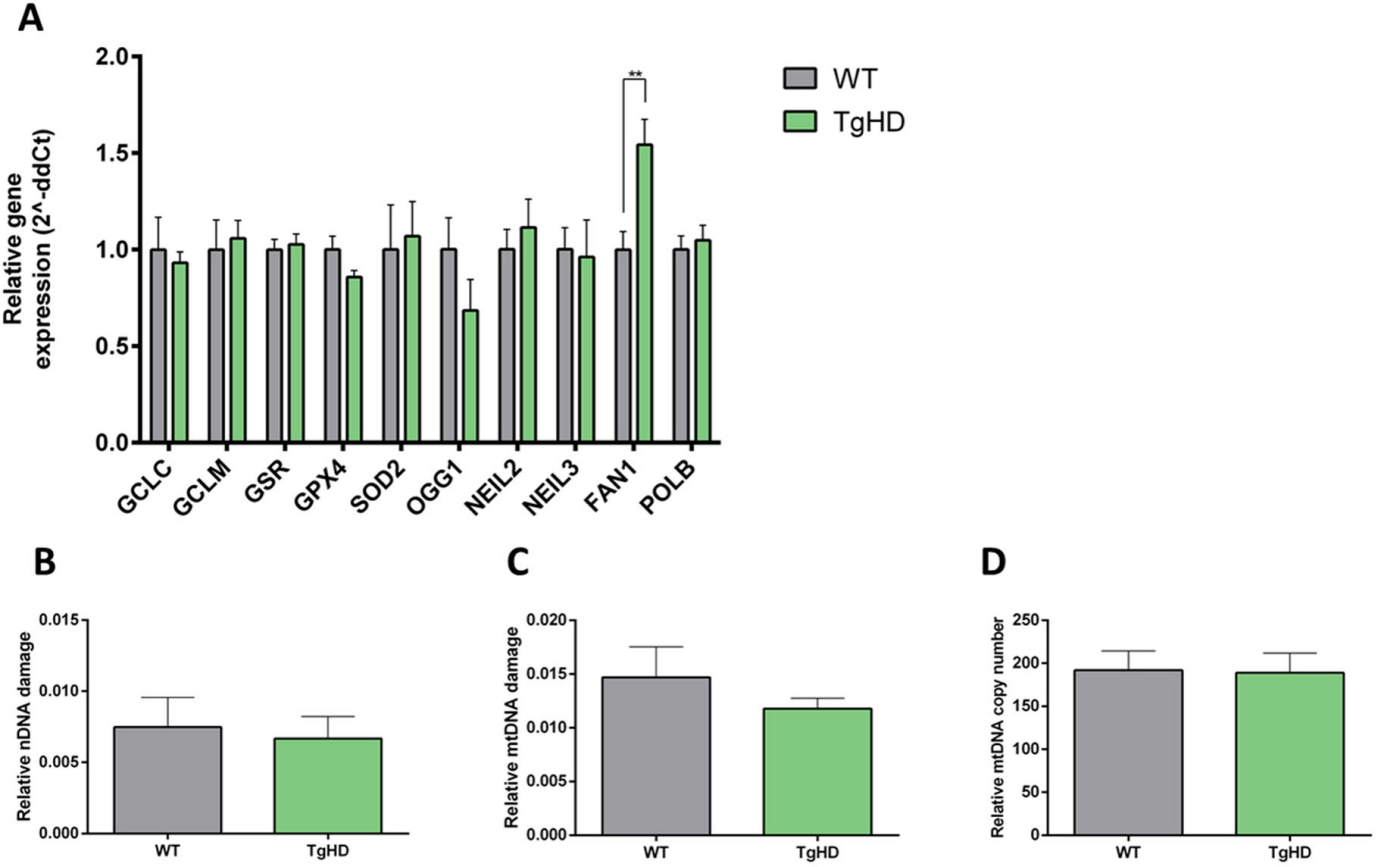
**Genes participating in antioxidant defence and DNA integrity.** (A) Analyses of expression of mRNA for antioxidant genes revealed a slight decrease in *GPX4* (*P*=0.1043), together with elevated nuclease *FAN1* (**P=0.0082) in TgHD iPSCs, normalised to WT iPSCs. *n*=6, WT; *n*=6, TgHD. (B) Nuclear DNA damage in WT and TgHD iPSCs. *n*=6, WT; *n*=6, TgHD. (C) Mitochondrial DNA damage in WT and TgHD iPSCs. *n*=6, WT; *n*=6, TgHD. (D) Mitochondrial DNA copy number in WT and TgHD iPSCs. *n*=6, WT; *n*=6, TgHD. Measurements were performed in a single technical replicate, with each sample loaded in triplicate. The final results were calculated as the mean value of these three analytical replicates. Student's *t*-test with Welch's correction. Error bars indicate s.e.m.

Among the genes participating in antioxidant defence and DNA damage repair, only *FAN1* showed significantly higher expression in TgHD iPSCs than in WT controls (*P*=0.0082) (Fig. 8A). The unchanged expression of other antioxidants or DNA repair genes indicates the absence of oxidative stress in TgHD iPSCs. Moreover, the lack of significant difference in DNA damage level and mtDNA copy number between TgHD and WT iPSCs (Fig. 8B-D) indicates that increase in *FAN1* is independent of DNA damage driven by oxidative stress.

An additional function of the FAN1 enzyme is interstrand DNA repair and repair of extrahelical extrusion formed in triplet repeats, which has a protective role in CAG expansion in HD cells (Deshmukh et al., 2021a,b; MacKay et al., 2010; Phadte et al., 2023; Smogorzewska et al., 2010; Wang et al., 2014; Yoshikiyo et al., 2010). In accordance, high *FAN1* expression could have a protective role in CAG triplet stabilisation in our proliferating TgHD iPSCs. The U20S cell line model expressing different exon 1 length variants revealed the importance of FAN1 expression in CAG stability alongside CAG length-dependent expansion (Goold et al., 2019). Similarly, human iPSCs and neurons with 109 CAG repeat also showed the CAG length-protecting role of FAN1 in these mitotic active and non-mitotic cells (Goold et al., 2019; McAllister et al., 2022). However, the possibility of higher *FAN1* expression caused by long CAG repeat in the TgHD iPSCs cannot be ruled out and needs further investigation to clarify its function in the TgHD iPSCs and other cell models of HD.

## MATERIALS AND METHODS

### Ethics

Animal experiments were approved by the State Veterinary Administration of the Czech Republic and performed at the Institute of Animal Physiology and Genetics (IAPG) of the Czech Academy of Sciences, under experimental protocol number 53/2015.

#### Primary embryonal fibroblasts

Porcine embryonal fibroblasts were isolated from 40-day-old embryonal (two TgHD and two WT) individuals and cultivated in Dulbecco's modified Eagle medium (DMEM), high-glucose GlutaMAX™ medium (Gibco, 10566016) with 10% fetal bovine serum (Sigma-Aldrich, F7524), 1% MEM Non-Essential Amino Acids Solution (Gibco, 11140050) and 0.1% gentamicin (Sigma-Aldrich, G1397) in flasks coated with 1% gelatin solution (Sigma-Aldrich, G2500).

#### Reprogramming of iPSCs

Reprogramming of porcine primary embryonal fibroblasts was performed using AMAXA 4D Nucleofector and P3 Primary Cell 4D-NucleofectorTM X Kit L (Lonza, V4XP-3024). Fibroblasts were cultivated until reaching ∼70% confluency. For every transfection reaction, 800,000 primary cells were used. Harvested fibroblasts were resuspended in 100 μl transfection solution (82 μl P3 Primary Cell Nucleofector Solution and 12 μl Nucleofector Supplement) together with 2 μg plasmid DNA: 1 μg pPB-CAG.OSKML-puDtk piggyBac transposon plasmid containing expression cassettes, CAG promoter-driven mouse variants of OSKML genes (*Oct4*, *Sox2*, *Klf4*, *c-Myc* and *Lin28*) and PGK promoter-driven puDtk (Yusa et al., 2009), and 1 μg pCMV-hyPBase pcDNA3-based expression vector of a hyperactive piggyBac transposase (Yusa et al., 2011). Transfection was performed using a program for mouse embryonic fibroblasts (CZ-167). Transfected cells were incubated in transfection solution for 5 min at 37°C and then transferred into fibroblast culture media without gentamicin and plated in two wells of a six-well plate pre-coated with 1% gelatin solution (Sigma-Aldrich, G2500). After 48 h, cells from each well were transferred to a 60 mm Petri dish coated with 1% Geltrex™ LDEV-Free, hESC-Qualified, Reduced Growth Factor Basement Membrane Matrix (Gibco, A1413301). The next day, medium was changed to KnockOut™ DMEM/F-12 medium (Gibco, 12660012) with 20% KnockOut™ Serum Replacement (Gibco, 10828028), 1% alanyl-glutamine solution (Sigma-Aldrich, G8541) and 0.1% 2-mercaptoethanol (Gibco, 31350-010). This medium was further supplemented with 10 ng/ml bFGF (Sigma-Aldrich, SRP4037), 10 ng/ml LIF (Sigma-Aldrich, LIF1010), 10 μM RepSox (Sigma-Aldrich, R0158), 10 μM CHIR99021 (Sigma-Aldrich, SML1046) and 1 μM PD0325901 (Sigma-Aldrich, PZ0162). Medium was changed every day, and transfected fibroblasts were left on the same dish without passaging until iPSC colonies emerged. iPSC colonies were then manually picked up and further propagated. We used a StemPro™ EZPassage™ Disposable Stem Cell Passaging Tool (Gibco, 23181010) for mechanical passaging. We generated, in total, six lines from two WT animals and six lines from two TgHD animals (three clones from each individual). All iPSC lines were expanded to passage 13 and maintained stable morphology without signs of spontaneous differentiation or major morphological changes.

#### Karyotyping

The iPSCs were seeded on six-well plates coated with Geltrex™, and, after reaching 50-60% confluency, they were treated with 0.4 µg/ml colcemid (Roche, 10295892001) diluted in medium for 2.5 h before collection by using TrypLE™ Express (Gibco, 12604) for 6 min, followed by hypotonisation in 0.075 M KCl and fixation (methanol: acetic acid, 3:1). Standard G-banding was performed on metaphase spreads with subsequent Giemsa staining, and 20 metaphases per sample were analysed.

#### Differentiation of three germ layers

iPSCs were seeded on a 24-well plate pre-coated with 1% Geltrex™. After reaching ∼60% confluence, the cultivation medium was changed to 75% cultivation medium and 25% differentiation medium with endoderm I, mesoderm and ectoderm factors from a Human Pluripotent Stem Cell Functional Identification Kit (R&D Systems, SC027B). After 24 h, the medium was changed to 50% cultivation medium and 50% differentiation medium with endoderm I, mesoderm and ectoderm factors. After 48 h, the medium was changed to 25% cultivation medium and 75% differentiation medium with endoderm II, mesoderm and ectoderm factors. After 72 h, the iPSCs were passaged by using TrypLE™ Express (Gibco, 12604) for 6 min, seeded to a LabTek II (Thermo Fisher Scientific, 154526) coated with 1% Geltrex™, and cultivated in differentiation medium for 24 h before fixation and staining according to the protocol. The next day, cells were incubated for 1 h at room temperature with secondary antibody (Alexa Fluor 555, Invitrogen, A21432) diluted 1:500, after washing with 1% bovine serum albumin in PBS.

#### Alkaline phosphatase live stain

The iPSCs and porcine primary embryonal fibroblasts were seeded on a Nunc™ Lab-Tek™ II Chambered Coverglass (Thermo Fisher Scientific, 155360) pre-coated with 1% Geltrex™. After reaching the confluent state (50-60%), they were stained with Alkaline Phosphatase Live Stain solution (Thermo Fisher Scientific, A14353) according to the protocol. The porcine primary embryonal fibroblasts were also stained with 5 µl/ml Hoechst 33342 (Thermo Fisher Scientific, H3570) in the last washing step before microscopy scanning.

#### Immunocytochemistry and microscopy

Immunofluorescence staining was performed on iPSCs at passage 10. Cells were grown for 3-4 days on a Nunc™ Lab-Tek™ II Chambered Coverglass (Thermo Fisher Scientific, 155360) pre-coated with 1% Geltrex™. After cultivation, cells were fixed with 4% paraformaldehyde (PFA) in PBS (pH 7.4). Following fixation, cells were permeabilised with Triton X-100, blocked with 2% goat serum in 0.1% Tween-20 in PBS, and then incubated overnight at 4°C with primary antibodies (anti-OCT3/4, Santa Cruz Biotechnology, sc5279, 1:50; anti-NANOG, Cell Signaling Technology, 4903, 1:50; anti-SOX2, Cell Signaling Technology, 3579, 1:400; anti-SSEA4, Sigma-Aldrich, MAB4304, 1:200) diluted in 0.2% goat serum and 0.1% Tween-20 in PBS. The next day, cells were incubated for 1 h at room temperature with secondary antibodies (Alexa Fluor 555, Life Technologies, A21424 and A21429) diluted 1:500, after washing with 0.1% Tween-20 in PBS. Then, the cells were incubated for 10 min with 1 μg/ml DAPI in PBS (Sigma-Aldrich, D9542) and washed with PBS before scanning.

Imaging was performed using a Leica TCS SP5 confocal microscope with an HC PL APO CS2 63.0×1.40 OIL UV objective. The pinhole was open to 1.38 Airy unit. Samples were scanned bidirectionally, at 400 Hz speed with line averaging 3, 12-bit resolution and 1024×1024 format. The same exposure setup conditions were used for each marker across all samples. Optical *z*-slices were obtained using a 1.0 μm step. For all images, a maximum-intensity *z*-projection was selected.

#### Western blot analysis

Cell lysates of iPSC pellets were obtained using RIPA buffer (Sigma Aldrich, 20-188), and protein concentration was determined by Pierce™ BCA Protein Assay Kits (Thermo Fisher Scientific, 23225). Then, 10 μg total protein was loaded onto 4-12% Bis-Tris Mini Protein Gels (Thermo Fisher Scientific, NP0322BOX). After electrophoresis, proteins were transferred to nitrocellulose membranes and stained with Ponceau S Staining Solution (Thermo Fisher Scientific, A40000278) to visualise total protein. The membranes were blocked in a 2% skimmed milk diluted in TBST buffer (20 mM Tris, 150 mM NaCl, 0.1% Tween-20) and incubated with primary anti-PDK1 antibody (Abcam, ab90444, 1:1000) overnight at 4°C. Detection was carried out with horseradish peroxidase (HRP)-conjugated secondary antibody (anti-rabbit, Jackson ImmunoResearch, AB10015282, 1:10,000) incubation for 1 h at room temperature. The antibody-bound PDK1 was visualised with Amersham™ ECL™ Prime (Cytiva, RPN2232SK) documented with a ChemiDoc device (Bio-Rad). The Image Lab software (Bio-Rad) was used for quantification. Total protein was visualised using Ponceau S staining (Thermo Fisher Scientific, A40000279) as a loading control, and imaged with the ChemiDoc system (Bio-Rad) using Image Lab software for quantification. After imaging, the membranes were washed three times with TBST and incubated for 30 min with 30% hydrogen peroxide to inhibit peroxidase activity (described by Sennepin et al., 2009). After the inhibition, membranes were washed three times with TBST and incubated with the anti-HTT antibody EPR5526 (Abcam, ab109115, 1:2000) overnight at 4°C. Then, detection was carried out with a secondary antibody (anti-rabbit, Jackson ImmunoResearch, AB10015282, 1:10,000) incubation for 1 h at room temperature. After imaging, the membranes were washed three times in TBST and stripped with mild stripping buffer (15 g/l glycine, 1 g/l SDS, 10 ml/l Tween-20, pH 2.2) two times, then washed two times in PBS and two times in TBST before staining with Ponceau S Staining Solution (Thermo Fisher Scientific, A40000278) to visualise total protein. The membranes were then blocked in 5% skimmed milk diluted in TBST buffer and incubated with primary anti-ND1 antibody (Abcam, ab233289, 1 µg/ml) overnight at 4°C. Then, detection was carried out with secondary antibody (anti-rabbit, Jackson ImmunoResearch, AB10015282, 1:10,000) incubation for 1 h at room temperature, as previously.

#### Gene expression analysis

Total cellular RNA was isolated from cell pellets using an RNeasy Mini Kit (Qiagen, 74104), and 2 μg RNA was reverse transcribed using a High-Capacity cDNA RT Kit (Thermo Fisher Scientific, 4368814) according to the protocol. SYBR Green (Thermo Fisher Scientific, A25780) and specific primers designed for *Sus scrofa* (Tables S1 and S2) were used in qPCR according to the manufacturer's protocol using a CFX96 Touch Real-Time PCR Detection System (Bio-Rad). Relative quantification of gene expression was calculated using 2^(−ddCt), with beta-actin as a housekeeping gene.

#### Nuclear DNA and mtDNA damage and mtDNA copy number estimation

DNA was isolated from cell pellets using a DNeasy Blood and Tissue Kit (Qiagen, 69504), following the manufacturer's protocol. DNA quantification was performed by spectrophotometric analysis (NanoDrop 1000, Thermo Fisher Scientific) and adjusted to the desired concentration for downstream analysis. DNA integrity analysis used the RADF (real-time qPCR analysis of damage frequency) method. According to the protocol, 6 ng and 30 ng of DNA [mtDNA and nuclear DNA (nDNA), respectively] were added to non-TaqαI- and TaqαI-containing reaction mixtures. Damage frequency was calculated based on the differences between non-TaqαI- and TaqαI-containing reactions using 2^−(Ct^Taq^-Ct^nt^). Primers designed for mtDNA damage (MT-RNR1) and nuclear DNA damage (NDUFA9) were used for this quantification (Table S3). mtDNA copy number was analysed in triplicate at total DNA of 12 ng and 6 ng. mtDNA copy number was calculated as an average of both DNA dilutions using the formula 2ΔCt [ΔCt=Ct(NDUFA9)−Ct(MT-RNR1)] as the relative number of mtDNA to nDNA.

#### iPSC genotyping and electrophoresis

Total cellular DNA was isolated by a DNeasy Blood and Tissue Kit (Qiagen, 69504). Q5 polymerase (New England Biolabs, M0491S) was used for the PCR reaction, with the recommended protocol, annealing temperature of 60°C and 100 ng DNA in the PCR reaction. Three types of primers for the WT and inserted lentiviral construct in the *HTT* gene (Table S4) were used. DNA isolated from the fibroblasts of the founders was used as a positive control for transgenic and WT lines. The electrophoresis was run on 1.8% agarose gel in TAE buffer (40 mM Tris base, 20 mM acetic acid, 1 mM EDTA) for 150 min at 90 V.

#### PDH activity

The basal activity of the PDH complex was measured in tetraplicates according to Sheu et al. (1981), as the production of 14CO_2_ produced by decarboxylation of [1-14C]-pyruvate (Perkin-Elmer) with incubation time of 20 min. Protein was estimated according to Lowry et al. (1951).

#### Statistics

Data from qPCR and DNA damage analysis were imported to GraphPad Prism 6.01. Measurements (*n*=6) were performed in triplicates, with the average used for Student's *t*-test with Welch's correction or ANOVA. Measurement of PDK1 protein level was performed in duplicates and ND1 protein level in a single measurement. Data were imported to GraphPad Prism 6.01 for Student's *t*-test with Welch's correction analysis. The average of two to four measurements of PDH activity were analysed in the same way. All results were considered statistically significant when *P*<0.05.

#### Use of AI tools

No AI tools were used for preparation of the manuscript or figures, or analyses.

## Supplementary Material

10.1242/dmm.052585_sup1 Supplementary information
