# Supplementary material for: Induced pluripotent stem cells from a transgenic minipig model of Huntington's disease reveal early metabolic changes
Source: Dis Model Mech. 2026 May 11;19(6):dmm052585. doi: 10.1242/dmm.052585 (PMC13225199; doi:10.1242/dmm.052585)
Supplement: Supplementary information [file dmm-19-052585-s1.pdf]

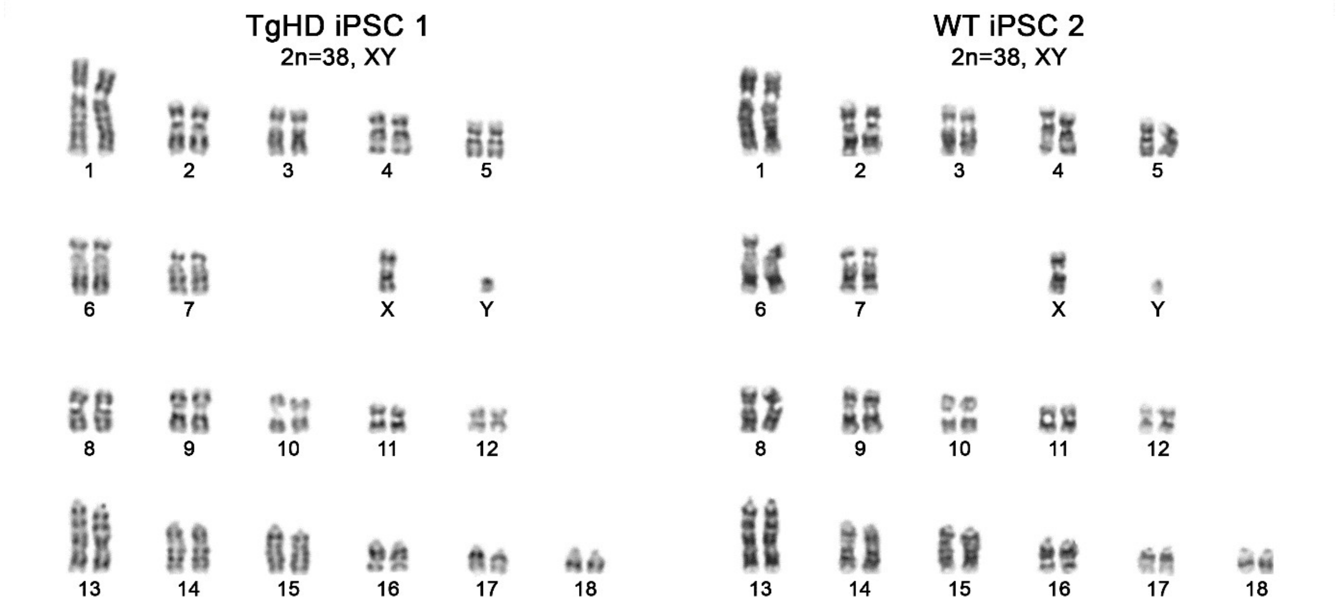


**Fig. S1. Karyotyping of iPCS lines used in experiments.** One representative karyotype is shown for each group.

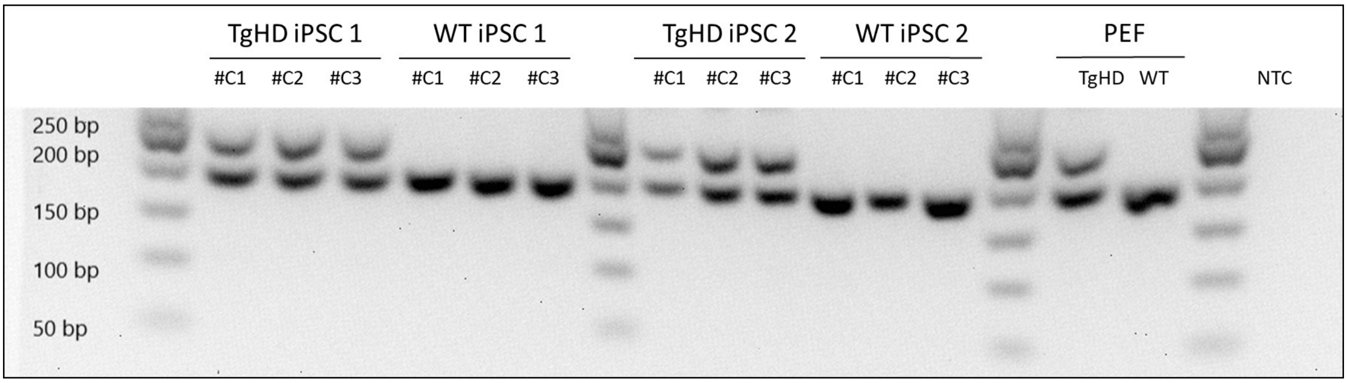


**Fig. S2. Genotyping of iPSC lines used in experiments.** The TgHD (1 and 2) and 6 WT (1 and 2) iPSC lines were used. Fibroblasts from TgHD and WT minipigs were used as a genotyping control and a non-template control for validation of the PCR reaction.

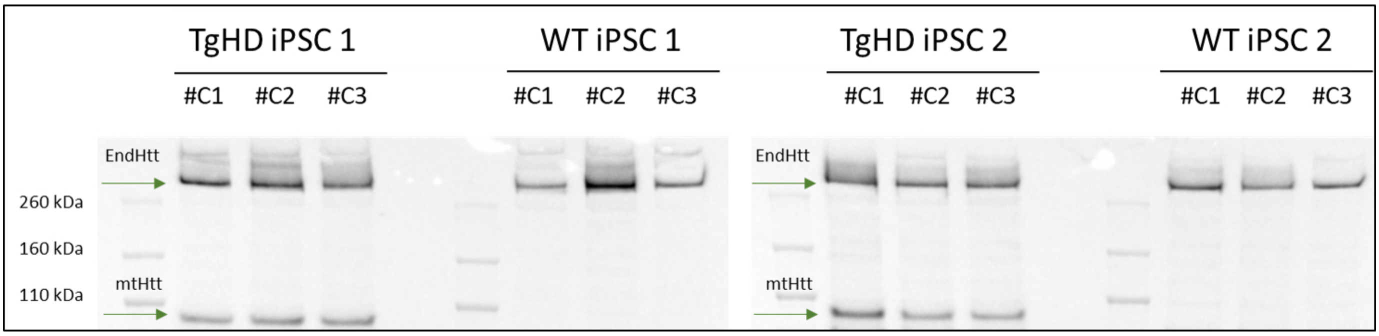


**Fig. S3. Detection of the mutant huntingtin protein expression.** The anti-huntingtin antibody (EPR5526) was used to detect the endogenous and mutant huntingtin in iPSC. The TgHD (1 and 2) and 6 WT (1 and 2) iPSC lines were used.

Disease Models & Mechanisms • Supplementary information

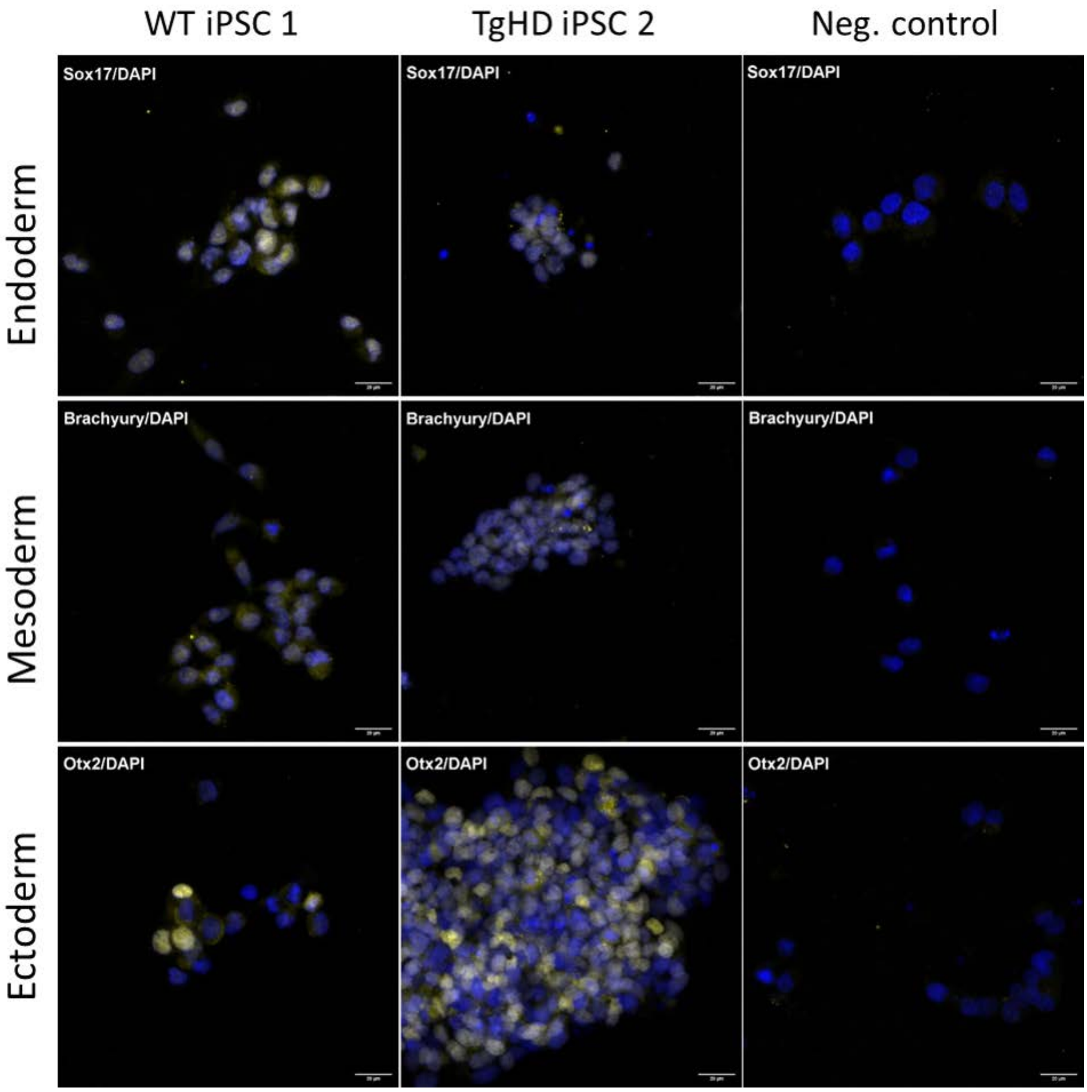


**Fig. S4. Differentiation potential of generated iPSC.** The representative pictures of differentiated marker Sox17 (endoderm), Brachyury (mesoderm), and Otx2 (ectoderm) expression in WT and TgHD iPSC. Scale bar 20 µm.

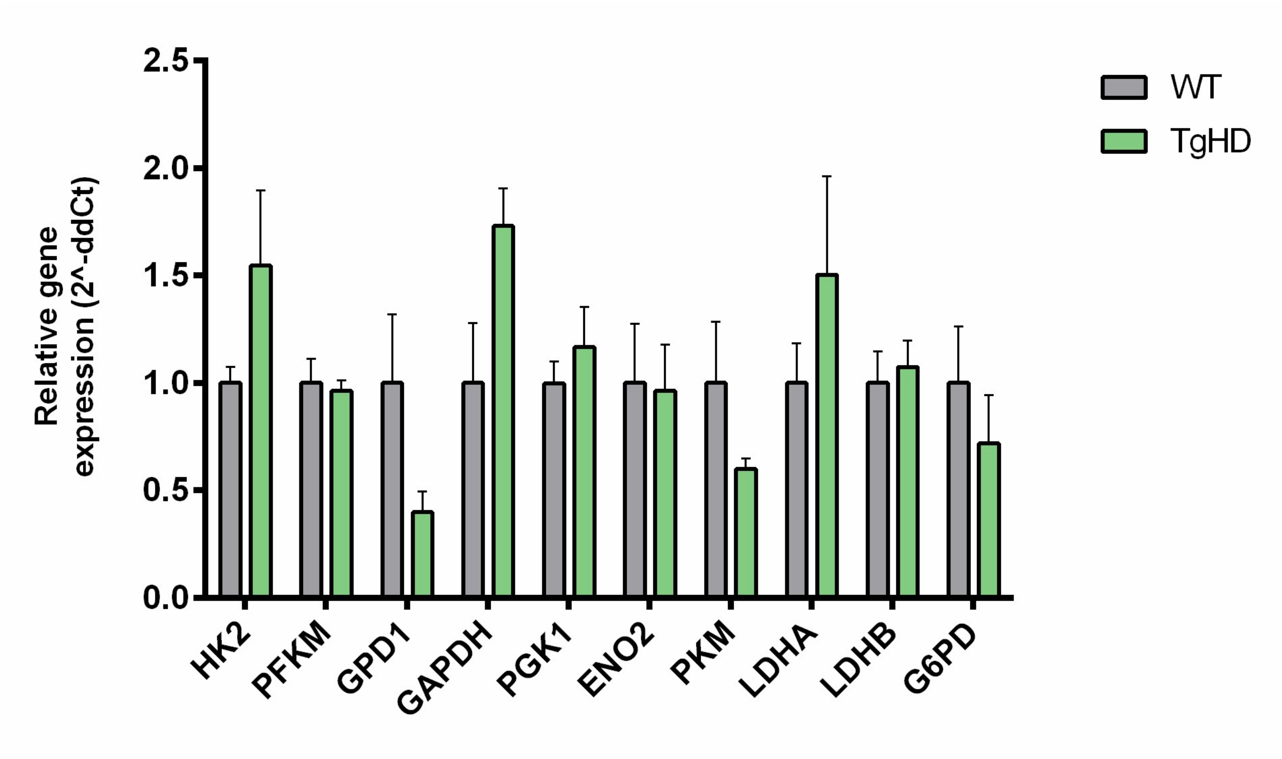


**Fig. S5. The expression of genes participating in glycolysis.** The graph shows the relative mRNA expression of glycolytic genes and G6PD. However, there is only a tendency of increased HK2 expression (p= 0.1833), GAPDH (0.0555), and lowered GPD1 (p= 0.1218), PKM (p= 0.2180) expression in TgHD iPSC (n= 6, WT, n= 6, TgHD, Student's t-test with Welch's correction). Error bars indicate SEM.
HK2= hexokinase 2, PFKM= phosphofructokinase, muscle type, GAPDH = glyceraldehyde-3-phosphate dehydrogenase, GDP1= glycerol-3-phosphate dehydrogenase 1, PGK1= phosphoglycerate kinase 1, ENO2= enolase 2, PKM= pyruvate kinase, muscle type, LDHA= lactate dehydrogenase A, LDHB= lactate dehydrogenase B, G6PD= glucose-6-phosphate dehydrogenase

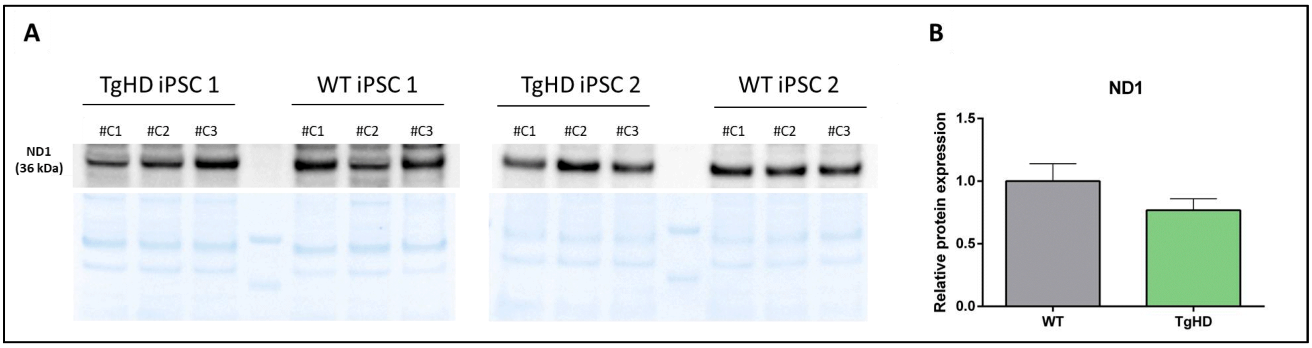


**Fig. S6. Protein level of ND1.** The anti-ND1 antibody (ab233289) was used for ND1 quantification in wild-type and transgenic iPSC on stripped membrane. The TgHD (1 and 2) and 6 WT (1 and 2) iPSC lines were used and normalised to MemCode. **A:** Western blot of ND1 and MemCode in iPSCs. **B:** The graph shows slightly decreased ND1 protein level in TgHD iPSC (WT, n= 6, TgHD, n= 6, p= 0.1979). Error bars indicate SEM.  ND1= mitochondrially encoded NADH dehydrogenase 1

Disease Models & Mechanisms • Supplementary information

**Table S1. List of primers used for gene expression analyses**

| *Target* | *Forward* | *Reverse* |
|---|---|---|
| *ACTB* | 5'-GAG AAG CTC TGC TAC GTC G -3' | 5'-CCA GAC AGC ACC GTG TTG G -3' |
| *NEIL2* | 5'-CGC CCA GGT CCA TGG AAA G -3' | 5'-CTC TGA CAG TGG GCT GCT AC -3' |
| *SOD2* | 5'-GCA AGG AAC AAC AGG TCT GG -3' | 5'-CGG CGT ATC GCT CAG TTA CA -3' |
| *LDHA* | 5'-CCT AAT GGG GGA AAG GCT GG -3' | 5'-CGC TCC ATA CAG GCA CAC T -3' |
| *PGK1* | 5'-ATG CTT CTG GGA GCA AGG TTA -3' | 5'-CGG TGA GCA GTA CCA AAA GC -3' |
| *HIF1a* | 5'-CAT CAG TTG CCA CTT CCC CAT -3' | 5'-CAA AAC CAT CCA AGG CTT TCA -3' |
| *VEGF* | 5'-GTG CCC ACT GAG GAG TTC AA -3' | 5'-GGC CCA CAG GGA TTT TCT TG -3' |
| *UCP2* | 5'-TAC AAA GGG TTC ACG CCC TC -3' | 5'-GCC CTC TTC AGC TGC TCA TA -3' |
| *UCP3* | 5'-ATT CCA GGC CAG CAT ACA CG -3' | 5'-GTC ACC ATC TCG GCA CAG TT -3' |
| *CYTB* | 5'-GTA GGC CTC AAT GTT CCT GCT -3' | 5'-TCG ACA TGG CCA ACA CAT CA -3' |
| *ND1* | 5'-AGC CAC ATC CTC AAT CTC C -3' | 5'-CCC GAT GAG TGC GTA TTT T -3' |
| *PDK1* | 5'-TGT GAA GAT GAG TGA CCG AGG -3' | 5'-AGG CGT GAT ATG GGC AAT CC -3' |
| *NEIL3* | 5'-GGG CCA AAA GCT TTA CGG ATT -3' | 5'-CAG AGC ATT CGT CCT CCC TG -3' |
| *HK2* | 5'-GCT CAA CCA TGA CCA AGT GC -3' | 5'-AAC TCT CCG TGT TCT GTC CC-3' |
| *OGG1* | 5'-GCT GTA GCT TCC TCA TGT GCC -3' | 5'-CTC AGA AGT CCC TTC ACA CCC -3' |
| *SIRT1* | 5'-TCC ATG GCG CTG AGG TAT ATT -3' | 5'-CAG TTC CTC CAG CTC GCA TA -3' |
| *NRF2* | 5'-TGG GAC ATA GCC AGT GAT AAA C -3' | 5'-AGA CAG GCC ACA GCA TAA TC -3' |
| *FAN1* | 5´- AAT CAC AGC ATG CTT TCA GGC -3´ | 5´-GAA GAG AAG CAA AGC GCT CC -3´ |
| *ACO2* | 5´- TGC ACA AAA TGG CGC CTT AC -3' | 5´- AGG CCG GTT CAG TCG CTT -3' |
| *TFAM* | 5´- AGC TCA GAA CCC AGA TGC AAA A -3' | 5´- ACC TGC CAG TCT GCC CTA TAA -3' |
| *LDHB* | 5´- GGC GAC CCT TAC AAG CCG -3' | 5´- CAT CCG TCA GAG ACT TTC CCA -3' |
| *KEAP1* | 5´- CGT GGA GAC AGA AAC GTG GA -3' | 5´- CAA TCT GCT TCC GAC AGG G -3' |
| *GPX4* | 5´- TGG ATG AAA GTC CAG CCC AAG -3' | 5´- GCT CAG CAC ACA CTT GTT GA -3' |
| *GSR* | 5´- TTG TTG GGG CCG GTT ACA TT -3' | 5´- GCC AGC ATT CTC CAG CTC TT -3' |
| *SDHB* | 5´- GTA GAG AAG GCA TCT GCG GC -3' | 5´- GGT GTC AAT TCT TCG GGT GC -3' |
| *G6PD* | 5´- GAA GCC CGA GCG TAC CAG -3' | 5´- AGA GAC AAG GGG AAA TGC GG -3' |
| *CS* | 5´- GAG CAA GCC AGA ATT AAG ACC -3' | 5´- CCA GCA GCC AAA ATA AGC C -3' |
| *PDP1* | 5´- ATG AGC ATC TCT GCC TTG CTT -3' | 5´- GAG CCG ACA ACA TCC ACA AC -3' |
| *ENO2* | 5´- GGG ATG GTG ACA AAC AGC GA -3' | 5´- ACA CAC CCA GGA TGG CAT TG -3' |
| *GCLC* | 5´- TTC CTG CAC ATC TAC CAC GC -3' | 5´- ACA TGT ATT CCA CCT CGT CGC -3' |
| *GCLM* | 5´- GGA CAA AAC CCA GTT GGA GC -3' | 5´- TCA CAC AGC AAG AGG CAA GA -3' |

Disease Models & Mechanisms • Supplementary information

**Table S2. List of primers used for gene expression analyses, continues**

| *Target* | *Forward* | *Reverse* |
|---|---|---|
| *PDK2* | 5´- AGC AGT TTC TGG ACT TCG GA -3' | 5´- ATG GTC CTC AGG GTC CTT GT -3' |
| *PDK3* | 5´- AGC AGA TCG AGC GCT ACT C -3' | 5´- CGA AGG GCG GTT AAG CAA AT -3' |
| *PDK4* | 5´- TCA GAC AGA GGA GGT GGT GT -3´ | 5´- GAC GAG AAA TTG GCA AGC CG -3´ |
| *SIRT2* | 5´- CCT GTT TCC GGT GCC GTC -3' | 5´- ATC GGA ATC GGA GTC CTG AGC -3' |
| *MDH1* | 5´- GTT TGG AAC CCC AGA GGG AGA -3' | 5´- CAG TGA GGT CCA TCT TCT CAC G -3' |
| *MDH2* | 5´- AAT GCC AAG GTA GCT GTG CT -3' | 5´- CCT TTC ACA GTC GCT CTG GT -3' |
| *GOT1* | 5´- CAA TGG CTG ACC GCA TTC TG -3' | 5´- ATT CAA CCT GCT TGG GGT TCA -3' |
| *GOT2* | 5´- ATG GGC TTA TAC GGT GAG CG -3' | 5´- CGT TGA CAG GAG GGT TGG AA -3' |
| *POLB* | 5´- AGC CAA AGC TGC TAC ATC GT -3' | 5´- GCT GGC AAA CAC CCA TGA AC -3' |
| *IDH1* | 5´- CAG TAC TGC GTT TCA TCG CTC -3' | 5´- CAC TGT CTT GCA GAG AAG CCT -3' |
| *IDH2* | 5´- AGC CTC TCA GCC CCT CTT AG -3' | 5´- TGT TCC TGC CTC ATG TCA CC -3' |
| *PFKM* | 5´- GGA GAG CTG AGA CTA TAA GAG TGG -3' | 5´- CCA GAG GTT AAC ACG GCG AT -3' |
| *PKM* | 5´- CCT GAT AGC TCG TGA GGC TG -3' | 5´- AGG TCT GTG GAG TGA CTG GA -3' |
| *PDP1* | 5´- ATG AGC ATC TCT GCC TTG CTT -3' | 5´- GAG CCG ACA ACA TCC ACA AC -3' |
| *GPD1* | 5´- ACA ACC CGC TTC TAG CTT CTC -3' | 5´- AAG TTC CTG CCC TCC AAA GAG -3' |
| *REST* | 5´-TCC GGC AAC AAA GAA AAG GAG -3' | 5´- ATT GCT CTG CTC GGC CTT C -3' |
| *PC* | 5´- CCA CAA CTT CAG CAA ACT CTT CAG -3' | 5´- CGC CAA GGG CAC TCA TAC A -3' |
| *ME1* | 5´- AAT GCC TTG TGC AGC CTT AG -3' | 5´- TTC TGA GAT TGG GGA AGT GG -3' |
| *SOX2* | 5’ – AAG AGA ACC CCA AGA TGC ACA ACT -3’ | 5’ – GCT TGG CCT CGT CGA TGA AC -3’ |
| *OCT4* | 5’ – GTT CTC TTT GGG AAG GTG TT-3’ | 5’ – ACA CGC GGA CCA CAT CCT TC -3’ |
| *GAPDH* | 5’ - TGC TCC TCC CCG TTC GAC -3’ | 5’ - ATG CGG CCA AAT CCG TTC -3’ |
| *NANOG* | 5’ - CAT CTG CTG AGA CCC TCG AC -3’ | 5’ - GGG TCT GCG AGA ACA CAG TT -3’ |
| *REX1* | 5’ - TTT CTG AGT ACG TGC CAG GC -3’ | 5’ - GAA CGG AGA GAT GCT TTC TCA GAG -3’ |
| *SALL4* | 5’ - ATC CAC CTC CGC TCC CAT ACC -3’ | 5’ - CGT TGC CTG CCG TCA TCT TGT -3’ |

Disease Models & Mechanisms • Supplementary information

**Table S3. List of primers used for DNA damage assay and mtDNA copy number**

| ***Target*** | ***Forward*** | ***Reverse*** |
|---|---|---|
| *NDUFA9* | 5'-GTT GTG AAT GGT GCT AAC TGC T-3' | 5'-ACC AGA GAC AAT AAA GCA GAG GAG-3' |
| *MT-RNR1* | 5'-TCG CAA CTG CCT AAA ACT CA-3' | 5'-GAA TTG GCA AGG GTT GGT AA-3' |

**Table S4. Primers used for genotyping transgenic lines**

| ***Target*** | ***Sequence*** |
|---|---|
| *WT HTT forward* | 5’-GCC TTG GCC AGT CAT TTT AAC C-3' |
| *WT HTT reverse* | 5'- ACT CTG GAA ACG CAC CAC GAT T-3' |
| *lentiviral LTR* | 5'-GGA ACC CAC TGC TTA AGC CTC A-3' |

**Table S5. List of used iPSCs in the study**

| | |
|---|---|
| WT iPSC 1 | Clone 1 (#C1) |
| | Clone 2 (#C2) |
| | Clone 3 (#C3) |
| WT iPSC 2 | Clone 1 (#C1) |
| | Clone 2 (#C2) |
| | Clone 3 (#C3) |
| TgHD iPSC 1 | Clone 1 (#C1) |
| | Clone 2 (#C2) |
| | Clone 3 (#C3) |
| TgHD iPSC 2 | Clone 1 (#C1) |
| | Clone 2 (#C2) |
| | Clone 3 (#C3) |
